# Therapy-Related Myeloid Neoplasms in Chronic Lymphocytic Leukemia and Waldenstrom’s Macroglobulinemia

**DOI:** 10.4084/MJHID.2011.031

**Published:** 2011-07-09

**Authors:** Francesca Ricci, Alessandra Tedeschi, Marco Montillo, Enrica Morra

**Affiliations:** Division of Hematology, Niguarda Ca’ Granda Hospital, Milano, Italy

## Abstract

Secondary myelodysplasia (MDS) and acute myeloid leukemia (AML) are frequent long term complications in Chronic Lymphocytic Leukemia (CLL) and Waldenström Macroglobulinemia (WM) patients. Although disease-related immune-suppression plays a crucial role in leukemogenesis there is great concern that therapy may further increase the risk of developing these devastating complications.

Nucleoside analogs (NA) and alkylating agents are considered appropriate agents in the treatment of both CLL and WM patients. Prolonged immunosuppression related to NA therapy and the incorporation of these agents or their metabolites into DNA, with potentially mutagenic action, leads to speculation that their therapeutic use might be responsible for an increased incidence of second cancer especially when combined with other DNA damaging agents like alkylating agents.

In this review the published studies considering the occurrence of secondary MDS and AML in CLL and WM patients are reported and the potential role of chemotherapeutic agents in leukemogenesis is discussed.

## Introduction:

Advances in the treatment of Chronic Lymphocytic Leukemia (CLL) and Waldenström Macroglobulinemia (WM) have resulted in higher response rates and more durable remissions. Chemoimmunotherapy has become the standard of care and the addition of rituximab to combination chemotherapy is associated with improved outcomes.^1^–^5^ As a result, the number of cancer survivors is rapidly growing and the late toxicities of treatment particularly therapy-related myeloid neoplasms (t-MN) have become a more important concern.

Some authors have reported an increasing incidence of these late complications over the years^6^ probably due to the better diagnostic criteria employed, increased age of population and widespread and successful use of drug combination in cancer patients.

A comprehensive review of reported myelodysplasia and acute myeloid leukemia (MDS/AML) rates concluded that up to 10% of patients treated for indolent non-Hodgkin’s lymphoma (NHL) using either conventional dose alkylating agents-based regimens or high-dose therapy develop MDS or AML within a decade of primary therapy.^7^ Based on these data the risk of therapy-related MDS/AML is an important consideration in the initial choice of therapy in these conditions.

In this review we have summarized the possible mechanisms of leukemogenesis, analyzed studies conducted to delineate the incidence of secondary MDS/AML in patients with CLL and WM and discussed the potential relationship between these events and treatment with NA and alkylating agents.

## Drugs Inducing Secondary Myeloid Neoplasms and Mechanism of Leukemogenesis:

Important discoveries have been made over recent years that have contributed to a better understanding of the pathogenesis of neoplasm and in particular of secondary AML/MDS. There are several lines of evidence showing that different mechanisms cooperate in leukemogenesis defining a multistep process.

Disease related immunodeficiency, resulting from both cell mediated and humoral-mediated immunity defects, has been postulated as a possible mechanism predisposing to second cancers in non Hodgkin lymphoma (NHL) and in particular in CLL and WM patients. B-cell impairment, low gammaglobulin levels and quantitative and functional defects of various subsets of T cells have all been documented in lymphoprolipherative diseases and are implicated in defective immune surveillance favouring second cancers. In line with this hypothesis is the documented incremented occurrence of malignant tumours in patients having a background of humoral immunodeficiency or abnormalities in T-cell function such as common variable immunodeficiency, ataxia-telangectasia, HIV disease.^8^,^9^ Furthermore, some authors underline the crucial role of the immune system in the oncogenesis reporting an increased risk of second cancers also in untreated CLL and WM patients.^10^,^11^

However, several studies have raised concerns about the role of therapy in the development of second malignancies and t-MN are a well recognized distinct entity in the 2008 WHO classification of tumors of hematopoietic and lymphoid tissues.^12^

Cytotoxic agents associated with this complication include alkylating agents, topoisomerase II inhibitors, ionizing radiation, antimetabolites and antitubulin agents.^12^,^13^ The WHO classification considers therapy-related MDS/AML as a unique clinical syndrome even though some cases may satisfy the morphological or cytogenetic criteria for other entities. Indeed, particular cytotoxic agents are associated with therapy-related MDS/AML with characteristic biological and clinical features.

Alkylating agents damage DNA either by methylation or DNA inter-strand crosslink formation. The latency between treatment and t-MN is generally long, between 5 and 7 years and may be preceded by a myelodysplastic phase. Most cases present deletion or loss of the long arm or total loss of chromosome 5 (5q−/−5) and/or deletion or loss of the long arm or total loss of chromosome 7 (7q−/−7).^14^–^16^

Topoisomerase II is an essential enzyme in eukaryotic cells and is thought to play an important role in DNA replication, transcription and cell division. This enzyme catalyzes the breaking and rejoining of both DNA strands. Topoisomerase II inhibitors block the enzymatic reaction through relegation and enzyme release, leaving the DNA with a permanent strand break. Multiple DNA-strand breaks lead to cell death. Both the antineoplastic effect and the leukemogenic effect are due to chromosomal break-age and this breakage, resolved by chromosomal translocation, is the cause of the leukaemic transformation. A shorter latency of approximately 2 years characterized t-MN secondary to topoisomerase II inhibitors. Balanced chromosome translocations frequently reported after exposure to this chemotherapeutic agents involve 11q23 (MLL) or 21q22 (RUNX1). However, others may also occur, for example t(8;21) or t(15;17) in the case of therapy-related acute promyelocytic leukemia (t-APL).^12^,^17^,^18^

Antimetabolites share structural similarities with nucleotides, and can be incorporated into DNA or RNA, causing inhibition of cell proliferation. Among antimetabolite agents fludarabine is the nucleoside analogs (NA) more frequently used in the treatment of indolent lymphoproliferative disorders[].^19^ The combination of fludarabine with cyclophosphamide or other DNA damaging agents may increase the risk of MDS/AML due to synergistic effects in the ability to produce DNA damage. Fludarabine inhibits DNA repair and augments the cytotoxic effect of DNA damaging agents such as cyclophosphamide.^20^ This enhancement of DNA damage may also affect marrow progenitor cells. Such a mechanism could explain the observed impairment of peripheral blood progenitor cell collection after prior fludarabine treatment and could predispose to an increased risk of therapy-related MDS/AML.^21^ High occurrence of abnormalities of chromosome 5 and 7 has also been described in MDS/AML secondary to NA therapy.^22^–^24^

In addition to typical genetic abnormalities, t-MN are characterized by molecular changes which may cooperate in the pathogenesis of the disease. FLT3 and p53 mutations are the most frequent described. Chimeric rearrangement of transcription factors, including AML1, CBFB, MLL or RARA and NPM1 mutations have also been reported.^25^

An important role in the multistep secondary leukemogenesis has been recently attributed to the epigenetic changes. Epigenetic mechanisms such as DNA methylation, post-translational modifications of histone proteins and remodelling of nucleosomes affect chromatin structure and contribute to define heritable changes in gene expression. The abnormalities in DNA methylation profile have been described more commonly in therapy-related rather than de novo MDS/AML.^26^

Since only a small fraction of patients exposed to cytotoxic therapy developed t-MN, one of the important questions in the field is what predisposes individuals to develop the disease. A large number of studies have offered evidence demonstrating the importance of genetic variation in key genes, which include those involved in drug metabolism, protection of cellular entities from damage and repair of DNA damage. Mutations in these vital genes are rare while much more common are genetic polymorphisms in detoxification genes and in genes belonging to the major DNA repair pathways.^18^

Although the multistep leukemogenesis mechanism has not been completely clarified, the important knowledge achieved in the last years in this matter has laid the groundwork to a risk adapted therapeutic approach. An adequate screening of individual susceptibility to develop t-MN could influence initial treatment strategies with the goal of decreasing the incidence of these serious complications.

## Waldenstrom Macroglobulinemia:

WM patients have a well documented increased risk of second cancers and in particular of secondary MDS/AML. Although disease-related immune-suppression has been suggested as one of the main causes of this increasing risk,^10^ the role of therapy in this important matter is under investigation.

Several studies have been conducted in Europe and in the United States to retrospectively examine the incidence of secondary MDS/AML in treated WM patients.

Alkylating agents and NA such as fludarabine and 2-chlorodeoxyadenosine (2-CdA) have been considered appropriate first line agents for the treatment of WM.^27^–^28^

Among patients receiving alkylating agents the incidence of such events ranged from 0 to 6% (Table 1) in different retrospective studies.^29^–^33^ Among them the most consistent data have been published by Ghobrial et al^33^ on 337 patients with newly diagnosed symptomatic WM. After a median duration of follow up of 11 years death occurred in 237 (70%) patients. The cause of death was due to WM or complications of therapy in 125 cases (37%) cases. Of these, death due to MDS/AML occurred in 16 patients (4.7%) indicating that this is a serious and fatal complication of alkylating agents therapy.

More recently attention has been focused on long term toxicity of NA-based therapy^34^,^35^ and retrospective comparative data has been reported by the French Cooperative Group on CLL/WM^36^ and by the Dana-Farber Cancer Institute.^37^

Leblond et al^36^ reviewed the incidence of secondary MDS/AML in 165 WM patients treated with NA agents in 3 trials performed from 1993 to 2003 by the French Cooperative Group on CLL/WM.

In the first trial^38^ 71 relapsed/refractory WM patients initially treated with alkylating agents received fludarabine. In the second trial^32^ 92 patients in first relapse after alkylating agents were randomized to receive fludarabine and CAP regimen (doxorubicine, cyclophosphamide, prednisone). In the third trial^39^ 49 patients with untreated or pretreated disease received fludarabine combined with cyclophosphamide.

The crude incidence of secondary MDS/AML varied from 1.4 to 8.9% in patients treated with fludarabine alone and was 6% in patients treated with fludarabine combined with cyclophosphamide. Incidence of such events were not different in the two groups of patients. More recently Leleu et al^37^ carried out a retrospective study to delineate the incidence of secondary MDS/AML in a large population of patients with WM and to determine the potential relationship between these events and treatment with NA. Among the 439 consecutive patients considered in this study 329 were treated with (193) or without (136) NA and 110 patients remained untreated. The median follow-up for all patients was 5 years. Overall 3 patients (1.6%) developed secondary MDS/AML among NA treated patients while no events were reported in both patients receiving other chemotherapeutic drugs and in untreated patients The 15-year probabilities of developing secondary MDS/AML were 8%. These data demonstrate an increased incidence of developing secondary MDS/AML among patients with WM treated with NA. The incidence of MDS/AML was not statistically different in patients treated with NA as first line treatment versus patients treated later in their disease history, in patients treated with either one or with multiple courses of NA, and in patients with history versus no history of treatment with an oral alkylating agent. Among the three patients who developed MDS/AML in this series, only one patient had previously received oral alkylating agents therapy. Furthermore, none of the clinical and laboratory features studied (gender, age, family history of haematological malignancies, markers of tumor burden, prognosis markers including WM-ISS) significantly predicted the occurrence of secondary MDS/AML events. However, no definite conclusion can be drawn because of the low number of events and therefore a lack of power in multivariate analysis might also explain that no risk factors except treatment with NA were observed.

Data reported in all these retrospective studies (Table 2) lead to conclude that although a role of NA in leukemogenesis has been wide recognized other data and prospective studies are needed to clarify the impact of fludarabine and alkylating agent combination compared to fludarabine alone in the increased risk of t-MN in WM patients.

In the last decade the use of rituximab has also been extensively evaluated in patients with WM and high activity of fludarabine and rituximab combination with or without cyclophosphamide has been demonstrated.

Treon et al^40^ reported the long term outcome of a multicenter prospective study examining fludarabine and rituximab association in 43 WM patients with less than two prior therapies. With a median follow-up of 40.3 months, 3 patients (7%) developed myeloid malignancies: MDS in one case and AML in two cases. Among them one had previously received chlorambucil and another had previously undergone high-dose chemotherapy with an autologous transplantation. The median time from protocol therapy to secondary MDS/AML was 39.4 months.

More recently toxicities data of fludarabine, cyclophosphamide and rituximab combination (FCR) have been reported by Leblond et al^41^ in 55 WM patients. Among them 40 had been previously treated with a median of 2 lines of therapy (range 1–4), including 24 patients with relapsed disease and 16 patients with refractory disease. After a median follow-up of 28 months secondary MDS/AML were observed in two heavily treated patients (3.6%). Similar results have been observed by Tedeschi et al in a group of 43 untreated and pretreated patients receiving FCR schedule: MDS occurred in three patients (6.9%), all of them previously heavily pretreated with alkylating agents, after 5, 24 and 60 months from FCR, respectively.^42^ Based on these data, addition of rituximab to fludarabine containing regimens does not seem to increase the incidence of t-MN (Table 2).

Although data on fludarabine treatment are more consistent, recent analysis of long term toxicity after 2-CdA therapy has also been published.

The MD Anderson Cancer Center performed a retrospective analysis of 111 consecutive, previously untreated patients with symptomatic WM who received 2 consecutive 4–6-week courses of either 2-CdA alone or in combination with other agents including prednisone, cyclophosphamide, and rituximab. Thirteen patients (12%) developed second malignancies, among them one patient had AML (0.9%). The median time to development of a second malignancy was 85.5 months.^43^

Laszlo et al^44^ recently reported the results with 2CdA and rituximab combination in 29 WM patients in first line therapy or previously treated without NA. With a median follow-up of 43 months no secondary MDS/AML was observed.

## Chronic Lymphocytic Leukemia:

Second malignancies are frequent complications in patients with CLL. The most frequent event is CLL transformation to large B-cell non-Hodgkin’s lymphoma also known as Richter’s syndrome.^45^–^47^ However, the secondary development of myeloproliferative disorders as well as solid tumors has also been documented in CLL patients.^48^,^49^

An increased incidence of these late complications has occasionally been reported in patients receiving alkylating agents. However, occurrence of therapy-related MDS/AML after exposure to alkylating agents seems to be lower when they are used intermittently as opposed to prolonged, continuous exposure.^50^

The study of French Cooperative Group on CLL reported data of two randomized trials in 1535 patients with previously untreated stage A CLL.^50^ In the first trial, 609 patients were randomly assigned to receive either daily chlorambucil or no treatment; in the second trial, 926 patients were randomly assigned to receive either intermittent chlorambucil plus prednisone or no treatment. Median follow-up for the first and second trials exceeded 11 and 6 years, respectively. CLL patients receiving either intermittent chlorambucil and prednisone or no treatment showed no increase of second tumors; in particular AML was observed in only one case (0.2%). However, a higher frequency of second cancers was reported in the group of patients treated with daily chlorambucil and AML occurred in 4 cases (1.3%).

No evidence of an increased risk of secondary MDS/AML was reported at M.D Anderson Cancer Center in a retrospective analysis of 1374 consecutive CLL patients, even though nearly three quarters of these patients had received prior treatment with alkylating agents.^51^ Authors suggest that the suppressive properties of disease–related cytokines acting on myeloid clones may be responsible for the lack of an increased rate of secondary AML/MDS in CLL despite prolonged treatment with alkylating agents and disease related immunosuppression.

In contrast to the published experience with alkylating agents, secondary MDS/AML are rarely reported following NA as monotherapy in CLL patients.^52^–^56^ However, the combination of NA with alkylating agents or other DNA damaging agents may increase the t-MN risk due to their synergistic effects (Table 3).

Morrison et al. presented data from intergroup CLL treatment trial comparing treatment with chlorambucil, fludarabine or chlorambucil plus fludarabine, in which a possible increase in the number of cases of therapy-related AML was observed.^57^ With a median follow-up of 4.2 years, six patients (1.2%) developed therapy-related MDS or MDS evolving to AML, from 27 to 53 months after study entry. This includes five (3.5%) of 142 patients treated with chlorambucil plus fludarabine and one (0.5%) of 188 receiving fludarabine. In the group treated with chlorambucil alone MDS or AML were not observed. These findings may indicate that alkylating agents combined with NA may increase the risk of therapy-related MDS/AML in CLL patients.

These data have been recently confirmed by the prospective randomized phase 3 trial, E2997, comparing FC with F alone as initial therapy in 278 CLL patients. With a median follow-up of 6.4 years, there have been 13 cases of t-MN, 9 cases after FC and 4 after fludarabine alone. The increased incidence of t-MN after FC, usually in the absence of additional treatment, suggests that FC is more leukemogenic than fludarabine.^58^

In contrast Robak et al.^45^ did not observed any MDS or AML in a retrospective series of 1487 CLL patients treated with 2-CdA, alkylating agents or both.

In the last five years chemoimmunotherapy regimens including rituximab showed high activity in CLL patients improving quality and duration of responses. The chemoimmunotherapy FCR has become a standard treatment for CLL based on the German CLL Study Group (GCLLSG) frontline CLL8 trial and the International REACH trial for patients in first relapse.^1^,^2^

Data of long term follow-up of FCR-therapy has been published by Badoux et al^59^ in a group of 284 previously treated patients followed in M. D. Anderson Cancer Center. Among them nine patients (3%) developed secondary MDS or AML (n=1) with a median of 20 months from starting FCR. Eight of these nine patients died after a median OS time of 23 months from FCR therapy. Comparable data has been reported by the same group^4^ with first-line FCR therapy: after a median follow-up of 6 years MDS occurred during first remission in 8 of 224 patients (2.8%) receiving immunochemotherapy.

More recently results published by Cancer and Leukemia Group Study^60^ suggest that incidence of t-MN does not increase with Fludarabine-rituximab combination (FR) without cyclophosphamide. After a median follow-up of 117 months none of untreated patients receiving fludarabine-rituximab concurrent or sequential combination developed t-MN during remission. Only one patient experienced therapy-related MDS, however, this occurred 45 months after FR treatment and 9 months after salvage treatment with FCR combination.

Studies examining consolidative autologous stem cell transplantation in CLL have also reported an increased risk of t-MN. Gribben et al identified in the Dana Farber series, an actuarial incidence of MDS/AML of 12% at 8 years.^61^ The 137 patients considered in this series received conditioning regimen with cyclophosphamide and total body irradiation. Previous debulking therapy consisted of alkylating agents and/or fludarabine and/or rituximab.

A lower incidence of secondary MDS/AML was reported by the German CLL-3 trial with three cases in 150 patients who underwent autograft.^62^

As well as the Dana Farber group a high occurrence of MDS/AML has also been described by Milligan et al in patients enrolled in the Medical Research Council Chronic Lymphocytic Leukaemia-5 trial.^63^ Of 115 newly diagnosed patients treated with fludarabine, 65 patients proceeded to autologous transplant. Conditioning was cyclophosphamide and total body irradiation in 49 (75%) patients and chemotherapy in 12 (18%). Ten patients developed MDS/AML; eight had undergone an autograft. Risk of MDS/AML was not associated with type of prior therapy. Five-year actuarial risk of developing MDS/AML postautograft was 12.4%. No analysed potential risk factor was predictive for MDS/AML development. Authors hypothesise that potential causative factors are fludarabine, low cell dose and transplant conditioning. The UK and German data differ in that there is an increased exposure to fludarabine and a lower infused cell dose in the UK. It has been suggested that the relatively low cell dose infused permitted a preferential expansion of damaged residual host stem cells, and that the myeloablative conditioning treatment will not destroy all hematopoietic cells with proliferative potential.

Recently results published of a phase 3 randomized trial of autografting in CLL versus observation for responding patients after first or second line treatment reported MDS in 4 patients: 3 in the autografiting arm (2.7%) and 1 in the observation arm (0.9%). The relatively low occurrence of MDS in the autotransplantation arm may be related to the limited follow-up of 5 years after randomization.^64^

## Conclusions:

The increased risk of secondary malignancies occurring in WM and CLL patients is well documented.

Overall published data reported frequently higher occurrence of t-MN in WM rather than in CLL patients, while in CLL patients transformation to Richter’s syndrome or the evolution to prolymphocytic leukemia is the more frequent long term complication.

The complex immunodeficiency resulting from B-cell impairment, hypogammaglobulinemia, T-cell dysfunction and decreased natural killer-cell activity, associated with these diseases may play a crucial role in leukemogenesis. However, there is great concern that therapy, especially with alkylating agents and purine nucleoside analogs, may further contribute to increase the risk of this devastating complication.

Although in the last few years several retrospective studies have been published on this topic, the real impact on risk of secondary MDS/AML complication of the single therapeutic agents has not been definitively clarified.

Indeed assessment of the specific risk is often confounded in patients with indolent lymphoprolipherative diseases by frequent exposure to multiple lines of cytotoxic therapy. Rates of secondary myeloid malignancies also vary with the length of follow-up and estimation may be falsely high if early reversible dysplastic features and cytopenia associated with cytotoxic treatment are misinterpreted.

Furthermore, the role played by additional immunosuppression due to the introduction of monoclonal antibodies in clinical practice needs to be better understood.

These considerations lead to conclude that the impact of secondary MDS/AML and the role of old and new drugs in the development of these late complications needs to be better evaluated in larger prospective studies, especially in young patients. In view of these data, we suggest that risk versus benefit should be considered when treating younger WM and CLL patients.

## Figures and Tables

**Table 1.** MDS/AML occurrence in WM patients treated with alkylating agents

**Authors**	**N° of patients**	**MDS/AML (N,%)**
Facon 1993^29^	167	2 (1.2%)
Kyle 2000^30^	46	3 (6.5%)
Garcia Sanz 2001^31^	217	3 (1.4%)
Leblond 2001^32^	46	2 (4.3%)
Ghobrial 2006^33^	337	16 (4.7%)
Leleu 2009^34^	136	0

**Table 2.** MDS/AML occurrence in WM patients treated with fludarabine–based regimens.

**Authors**	**Prospective study**	**N° pts**	**pretreated**	**schedule**	**Median follow-up time from study**	**MDS/AML (N,%)**
Leblond 1998^38^	no	71	yes	F 25 mg/m2 d1–5	34 mo	1/71 (1.4%)
Leblond 2001^32^	yes	45 45	yes	F 25 mg/m2 d1–5 Doxo 25 mg/m2 d1 CTX 750 mg/m2 d1 PDN 40 mg/m2 d1–5	34 mo	4/45 (8.9%) 2/45 (4.5%)
Tamburini 2005^39^	no	49	35 pts	F 30 mg/m2 d1–3 CTX 300 mg/m2 d1–3	43 mo	3/49 (6%)
Leleu 2009^37^	no	439	na	NA no NA untreated	5 y	3/193 (1.6%) 0/136 0/110
Treon 2009^40^	yes	43	17 yes	R 375 mg/m2/wk x 8 wks along with F 25 mg/m2/d x 5d x 6 cycles	40.3 mo	3 (7%)
Leblond 2010^41^	no	55	40 pts	F 40 mg/m2 os d1–3 CTX 250 mg/m2 os d1–3 R 375 mg/m2 d1	28 mo	2/55 (3.6%)
Tedeschi 2011^42^	yes	43	15 pts	F 25 mg/m2 d2–4 CTX 250 mg/m2 d2–4 R 375 mg/m2 d1	38.8 mo	3 (6.9%)
Rakkhit 2008^43^	no	111	no	2CdA 0.1 mg/kg/d iv d1–7 +/− PDN 60 mg/m2/d os d1–7 2CdA 1.5 mg/m2 sc x 3/d d1–5 + CTX 40 mg/m2 os x 2/d d1–7 +/− R 375 mg/m2/wk iv x 4 wks	55 mo	1 (0.9%)
Laszlo 2010^44^	yes	29	13 pts	2CdA 0.1 mg/kg/d sc d1–5 R 375 mg/m2 d1	43 mo	0

F=fludarabine; Doxo=doxorubicin; CTX= cyclophosphamide; PDN= prednisone; NA= nucleoside analogs; R= rituximab; 2CdA= 2-chlorodeoxyadenosine; mo=months; y= years; wk= week; d=day; os= oral; na= not applicable

**Table 3.** MDS/AML occurrence in CLL patients treated with NA–based regimens

**Authors**	**Prospective study**	**N° pts**	**pretreated**	**schedule**	**Median follow-up time from study**	**MDS/AML (N,%)**
Keating 1998^52^	yes	174	no	F 25–30 mg/m2/d d1–5 F 30 mg/m2/d d1–5 + PDN 30 mg/m2/d d1–5 F 30 mg/m2/d d1–5	98 mo 62 mo 39 mo	0
Leporrier 2001^53^	yes	924	no	FAMP 25 mg/m2 d1–5 *vs* CAP *vs* CHOP	70 mo	0
Johnson 1996^54^	yes	196	96 pts	F 25 mg/m2 d1–5 *vs* CAP	34 mo	0
Cheson 1999^55^	no	724	yes	F 25 mg/m2/d d1–5	7.4 y	0
Morrison 2002^57^	yes	544	no	F 25 mg/m2 d1–5 *vs* CHL 40 mg/m2 d1 *vs* F 10–20 mg/m2 d1–5 + CHL 15–20 mg/m2 d1	4.2 y	1/188(0.5%) 0 5/142 (3.5%)
Smith 2010^58^	yes	278	no	F 20 mg/m2 d1–5 + CTX 600 mg/m2 x d1 *vs* F 25 mg/m2 d1–5	6.4 y	9/141 (6%) 4/137 (3%)
Robak 2004^45^	no	1487	no	2CdA 0.12 mg/k/d d1–5 Alkylating agents: CHL, CHOP, COP 2CdA + Alkylating agents	2.6 y 3.3 y 4.4 y	0
Badoux 2011^59^	yes	284	yes	F 25mg/m2 d1/2–3/4 CTX 250mg/m2 d1/2–3/4 R 375–500mg/m2 d1	43 mo	9 (3%)
Tam 2008^4^	yes	224	no	F 25mg/m2 d1/2–3/4 CTX 250mg/m2 d1/2–3/4 R 375–500mg/m2 d1	6 y	8 (2.8%)
Woyach 2011^60^	yes	104	no	F 25mg/m2 d1–5 q4w x 6 cycles followed by R 375 mg/m2 x 4 doses *vs* F 25mg/m2 d1–5 + R 375 mg/m2 d1 followed by R 375 mg/m2 /wk x 4 doses	117 mo	0

F=fludarabine; Doxo=doxorubicin; CTX= cyclophosphamide; PDN= prednisone; NA= nucleoside analogs; R= rituximab; 2CdA= 2-chlorodeoxyadenosine; CHL= chlorambucil; CAP= doxorubicine, cyclophosphamide, prednisone; COP= cyclophosphamide, prednisone, vincristine; CHOP= doxorubicine, cyclophosphamide, prednisone, vincristine; mo=months; y= years; wk= week; d=day.
